# Adherence Tracking With Smart Watches for Shoulder Physiotherapy in Rotator Cuff Pathology: Protocol for a Longitudinal Cohort Study

**DOI:** 10.2196/17841

**Published:** 2020-07-05

**Authors:** David Burns, Helen Razmjou, James Shaw, Robin Richards, Stewart McLachlin, Michael Hardisty, Patrick Henry, Cari Whyne

**Affiliations:** 1 Division of Orthopaedic Surgery University of Toronto Toronto, ON Canada; 2 Holland Bone and Joint Program Sunnybrook Research Institute Sunnybrook Health Sciences Centre Toronto, ON Canada; 3 Working Condition Program Holland Orthopedic and Arthritic Centre Toronto, ON Canada; 4 Department of Physical Therapy University of Toronto Toronto, ON Canada; 5 Women's College Research Institute Toronto, ON Canada; 6 Joint Centre for Bioethics University of Toronto Toronto, ON Canada; 7 Mechanical and Mechatronics Engineering University of Waterloo Waterloo, ON Canada

**Keywords:** rehabilitation, treatment adherence and compliance, wearable electronic devices, machine learning, rotator cuff

## Abstract

**Background:**

Physiotherapy is essential for the successful rehabilitation of common shoulder injuries and following shoulder surgery. Patients may receive some training and supervision for shoulder physiotherapy through private pay or private insurance, but they are typically responsible for performing most of their physiotherapy independently at home. It is unknown how often patients perform their home exercises and if these exercises are performed correctly without supervision. There are no established tools for measuring this. It is, therefore, unclear if the full benefit of shoulder physiotherapy treatments is being realized.

**Objective:**

The proposed research will (1) validate a smartwatch and machine learning (ML) approach for evaluating adherence to shoulder exercise participation and technique in a clinical patient population with rotator cuff pathology; (2) quantify the rate of home physiotherapy adherence, determine the effects of adherence on recovery, and identify barriers to successful adherence; and (3) develop and pilot test an ethically conscious adherence-driven rehabilitation program that individualizes patient care based on their capacity to effectively participate in their home physiotherapy.

**Methods:**

This research will be conducted in 2 phases. The first phase is a prospective longitudinal cohort study, involving 120 patients undergoing physiotherapy for rotator cuff pathology. Patients will be issued a smartwatch that will record 9-axis inertial sensor data while they perform physiotherapy exercises both in the clinic and in the home setting. The data collected in the clinic under supervision will be used to train and validate our ML algorithms that classify shoulder physiotherapy exercise. The validated algorithms will then be used to assess home physiotherapy adherence from the inertial data collected at home. Validated outcome measures, including the Disabilities of the Arm, Shoulder, and Hand questionnaire; Numeric Pain Rating Scale; range of motion; shoulder strength; and work status, will be collected pretreatment, monthly through treatment, and at a final follow-up of 12 months. We will then relate improvement in patient outcomes to measured physiotherapy adherence and patient baseline variables in univariate and multivariate analyses. The second phase of this research will involve the evaluation of a novel rehabilitation program in a cohort of 20 patients. The program will promote patient physiotherapy engagement via the developed technology and support adherence-driven care decisions.

**Results:**

As of December 2019, 71 patients were screened for enrollment in the noninterventional validation phase of this study; 65 patients met the inclusion and exclusion criteria. Of these, 46 patients consented and 19 declined to participate in the study. Only 2 patients de-enrolled from the study and data collection is ongoing for the remaining 44.

**Conclusions:**

This study will provide new and important insights into shoulder physiotherapy adherence, the relationship between adherence and recovery, barriers to better adherence, and methods for addressing them.

**International Registered Report Identifier (IRRID):**

DERR1-10.2196/17841

## Introduction

### Overview

Rotator cuff pathology is the most common cause of shoulder pain and disability [1,2], and it is associated with significant utilization of health care resources [3] and societal and economic costs [1]. Exercise-based physiotherapy is the established first-line treatment for this condition [4-6] and is also a critical element of rehabilitation following rotator cuff surgery [7,8]. Adherence to prescribed physiotherapy is essential for successful rehabilitation in both conservatively and operatively managed patients [9,10]. However, adherence to physiotherapy is often poor [9,11], particularly in the home setting [9,12,13] and in worker populations [10]. Currently, there are no established methods to effectively and objectively measure adherence to home shoulder physiotherapy [12,14]. Thus, the rate and quality of participation with home shoulder therapy and the relationship between adherence and recovery are unknown.

We have developed the Smart Physiotherapy Activity Recognition System (SPARS), a novel solution for measuring adherence to home shoulder physiotherapy from inertial sensor data recorded on a smart watch using state-of-the-art machine learning (ML) techniques [15]. SPARS was shown to classify exercises for participation monitoring with high (99.99%) accuracy in 20 healthy adults.

The proposed study will further develop and validate our technology to assess home shoulder physiotherapy adherence to both participation and technique in a population with rotator cuff pathology. We will measure the rate of adherence, establish the relationship between adherence and recovery, and explore possible patient factors predictive of poor home physiotherapy adherence. In addition, we will examine ethical and related policy issues associated with the deployment of this technology, specifically investigating patient privacy, individual rights, clinical decision making, health resource allocation, and policy in the setting of artificial intelligence–based surveillance of patient self-management strategies. Guided by ethical analysis and a user-centered design process, we will develop and pilot test a conscientious rehabilitation program with individualized adherence-driven patient care.

### Rotator Cuff Pathology

Shoulder pain and dysfunction are among the most common musculoskeletal health problems and can affect 16% to 21% of the adult population with increasing incidence in advancing age [16-18]. Shoulder pain and dysfunction have an impact on the performance of essential activities of daily living (eg, dressing, hygiene, eating, and work) and result in substantial utilization of health care resources [3]. Rotator cuff pathology is the most common cause of shoulder pain and disability [1,2], accounting for up to 60% of all shoulder conditions [19]. Rotator cuff pathology is also a common cause of disability and employee time loss in the workplace and is associated with a high rate of claims and large societal and economic costs [1]. In the Ontario worker population, the shoulder is the second most frequent site of injury (after lower back) for high-impact claims [20].

Physiotherapy and nonnarcotic analgesia are the first-line treatment for symptomatic rotator cuff pathology [21]. Numerous studies and systematic reviews have concluded that many patients undergoing nonoperative rehabilitation for rotator cuff pathology achieve significant and durable improvements in shoulder pain and function [4,5,19,22-33]. The effectiveness of rehabilitation depends on the physiotherapy protocol that is used. Holmgren et al [34] demonstrated that a specific exercise protocol supervised by physiotherapists was superior to a self-directed range of motion exercises performed at home. Østerås et al [35] demonstrated a dose-response to rotator cuff rehabilitation, with high-dose (greater frequency and intensity) exercise training producing greater benefits than low-dose training.

Clinical trials have not demonstrated a clear benefit of surgical intervention over physiotherapy for the initial treatment of degenerative rotator cuff tears [22,23,36-38]. Current clinical practice guidelines, therefore, recommend that surgical management of rotator cuff pathology should be reserved for some selected cases (acute full-thickness traumatic tears in young patients) and when nonoperative modalities have failed to achieve the desired improvement in the function or symptoms after 6 to 12 weeks [24,39]. Postoperative rehabilitation with exercise-based physiotherapy is considered an essential component in the surgical management of rotator cuff pathology [7,8].

### Home Physiotherapy for Rotator Cuff Pathology

There is little comparative data on the relative effectiveness of supervised physiotherapy versus home-based exercise for rotator cuff pathology [3,24]. Although several trials have sought to address this question [34,40-42], all were of low sample size and adherence to the prescribed physiotherapy was not measured. The efficacy (or lack thereof) of a self-management strategy can only be determined if it is adhered to, which requires measurement of adherence. These studies also used different exercise regimens for their study groups, with the group in supervised therapy receiving a more extensive, specific, and individualized exercise regimen. To our knowledge, no study has directly compared the supervised and independent performance of the same rotator cuff rehabilitation protocol or sought to determine the optimal balance between supervised and independent exercise in hybrid programs. Despite these limitations, the efficacy of independent exercise for rotator cuff rehabilitation is supported by many studies with good outcomes that made extensive use of home exercise in their rehabilitation protocol [3,7,8].

### Adherence to Rehabilitation

Adherence is defined as the extent to which the patient follows medical instructions [43]. There is a general assumption that, where a treatment program is efficacious, adherence to the program improves outcome. Patient adherence to a prescribed physiotherapy has been shown to be an important predictor for the successful management of musculoskeletal disorders [9]. However, adherence to standard exercise protocols is often poor with estimates of non or partial nonadherence varying between 50% and 70% [9,11], with worse adherence for home exercise protocols [9].

In the context of physiotherapy and rehabilitation, the concept of adherence is multidimensional [44]. It includes behaviors such as attending clinical appointments, actively participating in physiotherapist-supervised rehabilitation activities, carrying out home exercise and rehabilitation activities, avoiding potentially harmful and contraindicated activities, and wearing protective or therapeutic devices [44]. Physiotherapy clinic attendance is readily measured, and the Sport Injury Rehabilitation Adherence Scale [44] has been used for evaluating in-clinic adherence. The correct use of protective devices has been measured by observations at clinical follow-up [10] and with temperature sensors [45-47].

Measuring adherence to home physiotherapy exercises remains an open problem [48]. Adherence to this component of a rehabilitation program is particularly vital as this activity calls for the greatest level of independent patient engagement in the rehabilitation process. Adherence diaries, in which patients log their independent exercises, is the recommended and most widely used measure of adherence to home exercise [48]. However, adherence diaries have significant limitations. The validity and reliability of the adherence diary have not been established, and poor patient acceptability results in low rates of diary completion [48]. Furthermore, the written diary has no means to establish if the prescribed exercises have been performed correctly. The proposed study will validate a novel method for measuring participation and technique adherence to home shoulder physiotherapy exercises.

### Adherence to Rotator Cuff Rehabilitation

Several studies on patients with rotator cuff pathology have measured rehabilitation adherence variables, including clinic attendance, adherence to postoperative restrictions, and home physiotherapy participation during rotator cuff rehabilitation [4,10,13,26,49-51]. Nonadherence (eg, to protective device use and clinic attendance) is more prevalent in workers’ compensation patients (52%) than nonworkers (4%) [10]. There is also a relationship between adherence and improved clinical outcomes in the nonoperative [50] and operative management [10,51] of rotator cuff pathology.

Unfortunately, adherence to home rotator cuff exercises has only been measured with adherence diaries [4,26,50] or in response to directed questioning [51]. The completion rates for the adherence diaries varied from 60% to 75% [4,26,50]. Furthermore, the validity of a patient’s self-reported adherence is questionable as a research instrument [52]. Therefore, the rate and effect of adherence to home rotator cuff exercise participation and technique remain unknown. The proposed study will address this knowledge gap in a cohort of 120 patients with rotator cuff pathology.

### Are Home Shoulder Physiotherapy Exercises Performed Correctly?

Patients with rotator cuff pathology have abnormal shoulder kinematics [53,54]. Due to the weakness of the rotator cuff, these patients develop a functional instability, where shoulder motion reduces the available supraspinatus muscle outlet, causing impingement [55]. There is a known relationship between altered shoulder kinematics, pain, and rotator cuff tear size [56]. It is believed that the altered kinematics and impingement exacerbate the condition, contributing to ongoing deterioration of the rotator cuff [54,55]. Restoration of normal shoulder kinematics, in particular muscular control of the scapula, is a focus of physiotherapy for rotator cuff pathology [19,57], and there are some data that contribute to improved patient outcomes [58]. An important role of the physiotherapist in rotator cuff rehabilitation is to supervise the process of kinematic retraining, ensuring that appropriate muscle recruitment and motion patterns are developed and maintained during exercise [59]. No study has determined whether the motion patterns trained during supervised therapy sessions are maintained in the home setting or independent exercise. The proposed study seeks to establish if there are kinematic differences between supervised and home rotator cuff rehabilitation exercises and determine if there is a relationship between exercise technique and shoulder recovery.

### Factors Affecting Adherence

The barriers to adherence to supervised outpatient physiotherapy clinics have been summarized in a systematic review by Jack et al [12] in 2010. There was strong evidence to suggest that low baseline levels of physical activity; low in-treatment adherence to exercise; low self-efficacy, depression, and anxiety; helplessness; poor social support; greater perceived number of barriers to exercise; and increased pain during exercise are all significant barriers to physiotherapy adherence. The majority of studies reviewed measured adherence only in terms of clinic appointments. Several studies [60-64] investigated the barriers to adherence to unsupervised home exercise programs. However, these studies suffered from the limitations of the existing adherence diary instrument outlined previously (poor objectivity, poor compliance with the diary, and inability to measure technique adherence). The barriers to unsupervised home physiotherapy, therefore, remain uncertain; they will be explored in the proposed study.

### Potential Technologies for Measuring Adherence to Home Shoulder Physiotherapy

Several technologies have been developed and pilot tested to provide more objective and complete assessments of adherence to home physiotherapy [13,15,65-72], but none have been validated in a clinical population. The common premise underlying a technical solution to adherence monitoring is using sensors to record patient home physiotherapy and have a computer algorithm classify activity type and potentially evaluate the technique. Various sensors have been investigated for this application, including video and depth sensors [65-68], strain sensors mounted on elastic bands [13], and inertial sensors [15,69-72].

Inertial sensors include accelerometers, gyroscopes, and magnetometers. These sensors are combined in small, inexpensive integrated circuit packages called inertial measurement units (IMUs). IMUs are found in smartphones, smart watches, and GPS devices, and they enable ongoing navigation with loss of GPS signals (eg, indoors). Advances in the capability of these sensors have enabled their use in demanding clinical applications, such as upper extremity motion tracking [73], clinical shoulder evaluation [74-77], kinematic analysis [78-82], and shoulder physiotherapy adherence evaluation [15,69,70].

Inertial sensors are, in our opinion, an ideal method for measuring adherence to home shoulder physiotherapy. These sensors are already integrated into robust, accessible devices, such as smart watches, facilitating low-cost deployment. Such wearable devices are unobtrusive and easy to use anywhere, unlike solutions based on video capture. Finally, they are highly accurate for this task [15] and can theoretically be used to measure adherence to any exercise involving motion.

Several studies have investigated the potential for shoulder physiotherapy adherence evaluation using inertial sensors in the laboratory setting. McGirr et al [69] used a 3-axis gyroscope to classify 3 shoulder exercises performed with an elastic band and were able to classify activity type, but with relatively low accuracy (86%-91%). Pan et al [70] used classical ML techniques to classify 5 different shoulder exercises from multiple synchronized inertial sensors mounted on the chest, upper arm, and wrist, achieving classification accuracy of 96.9% in the laboratory setting. Our pilot study used state-of-the-art deep learning algorithms to classify 7 evidence-based physiotherapy exercises from a single wrist-worn IMU in a commercial smart watch with an accuracy of 99.99% [15].

Deep learning is a class of ML algorithms for training deep hierarchical models, such as artificial neural networks (ANNs), which are ML models partly inspired by biological neural networks found in animal brains [83]. Recent advances in deep learning and computer performance have together spurred revolutionary developments in the capabilities of modern artificial intelligence systems [84]. In our pilot work, we demonstrated that deep learning significantly outperforms classical ML algorithms for inertial exercise classification [15]. Unlike classical ML models, neural networks can also benefit from transfer learning [85], which facilitates network retraining and updating in various contexts, such as adding a new exercise class or patient to the training dataset. Although significant computational resources are required for training ANNs, trained ANNs can be compact and deployable with modest computer hardware [86]. The ML models we developed in our pilot work were designed for deployment and real-time operation on a smart watch, having only 300,000 parameters. This aspect of the SPARS design permits a highly reliable edge-computing system architecture, where end-user apps are always responsive and function independent of internet connectivity or cloud computing infrastructure.

### Ethical and Policy Issues Related to Measuring Adherence to Patient Self-Management Strategies

There are a number of ethical and policy issues raised by the proposed project that will be examined in detail, concurrent with the clinical validation of the SPARS technology. Building on our past work addressing the challenges of introducing innovative digital health technologies [87], this analysis will inform the design and use of SPARS in the delivery of adherence-driven care. Drawing on the critical bioethics of Hedgecoe [88], we will examine the actual lived experiences and policy realities that are implicated in SPARS deployment and program development. However, we can anticipate, based on the bioethics literature, that issues at the intersection of ethics and policy will be raised in 3 distinct areas.

First, the act of surveillance introduces a number of ethical challenges. One is that surveillance stands to influence the behavior of participants, despite the conflicting evidence on the ways in which the proposed *Hawthorne effect* might occur [89]. Beyond a simple form of data collection, however, the collection of ubiquitous data about movements could have a number of unanticipated consequences [90]. A potential effect is to enhance the frequency or quality with which exercises are performed, but others include the distrust of the technology and health care providers who ultimately observe the data. Where participants might feel coerced into completing a form of physical activity, especially in cases where workplace injuries are involved, resistance might be substantial [91]. These are ethically important challenges that need to be addressed throughout this program and will dictate what data are collected, how they are controlled, and how and to whom the analysis results are presented.

Second, the surveillance and related project data promise to generate insights about which types of patients engage in exercises with the greatest fidelity. Findings related to patient profiles of those who engage and those who do not could be used in a variety of ways, including reallocating resources to those who stand to benefit from high engagement or enhanced behavioral change programs for those who do not engage. Generally, these questions of resource allocation relate to the priority-setting process for rehabilitation programs [92] and require detailed analysis to inform policy.

Third, the algorithms being deployed in this study are developed by individuals with particular views of the world. The types of data being used to train the algorithms, assumptions about the functioning of the technology, and beliefs about appropriate outputs are all shaped by the education, race, gender, and other social realities of the people writing the computer code. These points relate to bias that could potentially be built into the algorithm itself and the fairness of its outputs [93].

Each of these points will form a substantive focus on the ethical and policy analyses that are part of this study.

### Specific Research Aims

The specific research aims, and related objectives and hypotheses for this study are detailed below.

#### Aim 1: Develop and Validate the Smart Physiotherapy Activity Recognition System For Evaluating Shoulder Physiotherapy Adherence

##### Objectives

The related objectives are as follows:

1. Inertial sensor data are collected during supervised physiotherapy for 120 patients with rotator cuff pathology over full treatment duration (up to 5 months).
2. Technical failures preventing supervised data collection are less than 5%.
3. The SPARS artificial intelligence is trained to classify physiotherapy exercise type and technique with classification accuracy exceeding 90% and 80%, respectively.

#### Aim 2: Measure the Rate of Adherence to Home Shoulder Physiotherapy, the Relationship Between Adherence and Recovery, and Identify Barriers to Home Physiotherapy Adherence

##### Objectives

The related objectives are as follows:

1. Inertial sensor data are collected during home physiotherapy for 120 patients with rotator cuff pathology over full treatment duration (up to 5 months).
2. Technical failures preventing home data collection are less than 5%.
3. Clinical outcomes: return to work status, shoulder range of motion, rotator cuff strength, and patient-reported outcomes of pain and disability are collected pretreatment, monthly through treatment (up to 5 months), and at 12-months final follow-up. The final follow-up exceeds 85%.
4. A total of 23 potentially significant predictors of home physiotherapy adherence (identified in Methods: Barriers to Adherence) are collected for all study participants at the time of recruitment.

##### Hypotheses

The related hypotheses are as follows:

1. Exercise technique adherence is better for supervised sessions than at home (*P*<.05).
2. There will be a statistically significant association (*P*<.05) between home physiotherapy exercise participation and technique adherence and the identified clinical outcomes.

#### Aim 3: Develop and Pilot Test a Conscientious Smart Physiotherapy Activity Recognition System–Powered Shoulder Rehabilitation Program That Provides Individualized Adherence-Driven Patient Care in Accordance With Ethical Innovation and User-Centered Design Practices

##### Objectives

The related objectives are as follows:

1. Conduct and analyze qualitative interviews with stakeholders and end users to identify functional needs and different perspectives on ethical commitments and challenges related to individual experiences, privacy, clinical decision making, health resource allocation, and policy.
2. Develop novel shoulder rehabilitation strategies within the Working Condition Program, leveraging validated SPARS adherence analytics to conscientiously guide physiotherapy treatment as per the ethical, policy, and user-centered design analysis.
3. Pilot test the rehabilitation program in 20 patients. Conduct postimplementation qualitative interviews to re-examine ethical, policy, and design issues.

##### Hypotheses

The methodology to be drawn upon for the service design process and qualitative evaluation is fundamentally inductive and user-driven. Therefore, it would not be appropriate to articulate specific hypotheses to drive our data collection. We have outlined several ethical and policy issues that we will address, but we will approach the participants in ways that encourage them to describe the experiences and issues most pertinent to their own viewpoints. In so doing, we will remain open to all insights that emerge from design activities and qualitative interviews.

## Methods

### Smart Physiotherapy Activity Recognition System Technical Development and Clinical Validation Study (Aims 1 and 2)

We will further develop SPARS to evaluate the physiotherapy exercise technique and validate the SPARS exercise type and technique evaluations in a clinical population undergoing physiotherapy treatment for rotator cuff pathology. The relationship between adherence and recovery and barriers to home physiotherapy adherence will also be determined in this clinical validation study.

#### Population

We will conduct a prospective longitudinal cohort study of 120 patients who have been referred to Sunnybrook Health Sciences Centre for the management of rotator cuff pathology. The inclusion criteria will be as follows: (1) age ≥18 years; (2) diagnosis of unilateral rotator cuff tendinosis, shoulder impingement syndrome, or degenerative or traumatic rotator cuff tear; (3) planned conservative or operative management; and (4) capacity to participate in home shoulder physiotherapy. Exclusion criteria will be as follows: (1) upper extremity neurologic deficit; (2) bilateral symptomatic rotator cuff pathology; and (3) failed surgical management of rotator cuff pathology.

#### Inertial Data Collection

Each study subject will be provided with a smart watch to be worn on their affected extremity when performing the prescribed physiotherapy exercise. The smart watch must be worn for all physiotherapy exercise sessions conducted over the duration of treatment, up to a maximum of 5 months. This includes all supervised physiotherapy sessions at the Holland Centre and any physiotherapy exercise sessions conducted by the patient independently (home exercises). No alteration to the prescribed physiotherapy will be undertaken during this clinical validation study.

The smart watches purchased for this study will have a 9-axis IMU (3-axis accelerometer, 3-axis gyroscope, and 3-axis magnetometer). The inertial sensors will be sampled at 50 Hz and recorded to the internal memory storage on the smart watch. The inertial data will be encrypted using the Rivest–Shamir–Adleman public-key cryptosystem. The smart watch will permit up to 150 hours of sensor data recording to its 4 GB internal memory. The sensor data will be retrieved and removed from the smart watch when the patient attends supervised physiotherapy at the Holland Centre (2 times per week).

#### Inertial Data Labeling

The researchers will attend a supervised physiotherapy session for each patient once every 2 weeks to record exercise type and technique labels synchronously with inertial data collection, based on the input from the treating physiotherapist. The technique will be labeled as a binary variable, indicating if the treating physiotherapist was satisfied that the exercise was performed correctly (as intended). The treating physiotherapist will also make a record of prescribed home exercises and exercise type and technique evaluations at each supervised physiotherapy session.

#### Supervised Physiotherapy Video Recording

Color video recordings of supervised physiotherapy sessions will be made during 30 supervised inertial data collections. The videos will be used for the reliability analysis of the exercise type and technique labels assigned by the treating physiotherapist, and 2 blinded physiotherapists will review and reclassify the video data independently.

#### Home Physiotherapy Adherence Diaries

Patients will log their home physiotherapy activities in an adherence diary for 2 weeks, during the strengthening phase of their rehabilitation. Validation of the SPARS adherence measurement will include a comparison with these data.

#### Clinical and Return to Work Outcomes Data

Validated clinical outcomes will be collected to measure the relationship between home physiotherapy adherence and patient recovery. The following validated clinical outcome measures will be collected (1) pretreatment; (2) monthly through treatment; and (3) at 12-months final follow-up (items 1-3 only); these items are routinely collected by the Sunnybrook’s Working Conditions Program (WCP):

1. Work status (full-time, part-time, off-work, modified, or regular duties)
2. Numeric Pain Rating Scale (NPRS)
3. Disabilities of the Arm, Shoulder, and Hand (DASH) scores
4. Rotator cuff strength testing using manual muscle testing
5. Shoulder active range of motion measured with a goniometer.

The NPRS is valid and reliable for clinical practice [94,95]. The DASH has proven reliability, validity, and responsiveness in patients with shoulder problems [96-98]. Manual muscle testing and range of motion measurement using a goniometer are standard accepted methods for rotator cuff strength testing and shoulder range motion evaluation in the clinical setting [99], with established validity and reliability [100-104].

#### Barriers to Adherence

The following potentially significant adherence predictors [12,105-110] will be collected for each patient at recruitment: age, sex, body mass index, dominant side involvement, symptom duration, mechanism of injury (traumatic or degenerative), rotator cuff tear thickness and size, type of operative procedures, comorbidity (cumulative illness rating scale [111]), smoking status, alcohol intake, opioid intake, cannabinoid intake, the baseline level of physical activity, education, marital status, job demands, socioeconomic status (current income), perceived social support (Enhancing Recovery in Coronary Heart Disease Social Support Inventory [112]), patient self-efficacy (2-item Pain Self-Efficacy Questionnaire [113]), Patient Expectation Questionnaire [114], and Hospital Anxiety and Depression Scale [115].

#### Data Management, Monitoring, and Reporting

The data for this project consist of the following (1) inertial sensor data; (2) video data; (3) clinical outcome scores; (4) adherence predictor variables; and (5) adherence diaries. Items 2-4 include personal information. Video data will be anonymized with face deidentification and audio removal. Data items will be linked with a subject identification number and stored on secure servers at Sunnybrook Research Institute, behind institutional firewalls, with restricted physical access. The database will be encrypted, and access will be limited to researchers carrying out activities related to the proposed study. Continuous data redundancy and versioning will mitigate the risk of large data loss.

When a patient attends supervised physiotherapy at the Holland Centre, their smart watch inertial data are uploaded to Google Cloud Storage. The retrieved data are immediately verified by a software tool that checks and reports the proper functioning of the sensors and correct watch usage to the researchers, ensuring that anomalies are detected promptly. Our research engineer will administer the study database, review the incoming data, and update the research team during monthly project meetings.

#### Smart Physiotherapy Activity Recognition System-Home Physiotherapy Participation Adherence

The ML algorithms underlying SPARS will be trained in a supervised learning framework to classify (recognize) different activities from the characteristic time series of inertial signals they produce in the smart watch inertial sensors. A detailed description of our implementation and its evaluation are presented in our previous pilot study [15] and our open-source software package for ML time series (seglearn) [116]. Briefly, sensor data are segmented into fixed-length windows (2-4 sec in length) that are learned by a deep convolutional recurrent neural network (CRNN) classifier. Once trained, the classifier can be used to reconstruct a record of activities from the recorded inertial sensor data.

The ML algorithms underlying SPARS will be trained and validated using the labeled inertial sensor data collected during supervised physiotherapy sessions. The trained SPARS algorithms will then generate a record of exercises performed from inertial sensor data collected during unsupervised home sessions and supervised sessions during which synchronous data labeling was not performed. Comparing the quantity and frequency of home exercises measured by SPARS with the prescribed exercises will serve as our measure of participation adherence. Participation adherence will be measured as a percentage of the prescribed activity over each 2-week interval of treatment.

#### Smart Physiotherapy Activity Recognition System-Home Physiotherapy Technique Adherence

A second supervised learning algorithm will be trained, validated, and optimized to classify the exercise technique from the labeled inertial sensor data collected during the supervised physiotherapy sessions. We anticipate that a CRNN architecture similar to the exercise type classifier will likely be suitable. The validated technique classifier will be used to evaluate the technique adherence from the inertial data collected during unsupervised home sessions.

Anticipated challenges with the proposed supervised learning approach to technique evaluation include class imbalance and recovery-related changes in exercise performance. It is anticipated that many more correct technique training examples may be available for some exercises and that an exercise may be performed incorrectly in ways not observed during the supervised sessions, leading to class imbalance. It is also anticipated that through recovery, the expectation for technique and performance of an exercise may change over time, creating a moving target for the technique classifier. The challenge of class imbalance may be mitigated using several potential approaches including (1) random over and undersampling [117]; (2) cost-sensitive learning [117]; (3) semisupervised learning [118]; (4) anomaly detection [119,120]; and (5) synthetic data augmentation [121]. The challenge of recovery-related performance changes may be mitigated by providing our technique classifier metadata related to the classification task (eg, treatment week, pathology, and surgery) [116], implementing patient-specific ML models using transfer learning, and periodically retraining the ML models through recovery.

### Smart Physiotherapy Activity Recognition System–Powered Rehabilitation Program Development and Pilot Testing (Aim 3)

We will examine functional needs and ethical and related policy issues associated with the deployment of SPARS to design, implement, and pilot test novel rehabilitation strategies that provide individualized adherence-driven patient care in accordance with ethical innovation and user-centered design practices.

#### Examination of User Needs, Ethical Issues, and Policy Issues

We will complete qualitative interviews with patients, health care providers (primary care physicians, orthopedic surgeons, and physiotherapists), and policy-level stakeholders at the WCP, Workplace Safety and Insurance Board (WSIB), and the Ontario Ministry of Health and Long Term Care to examine different perspectives on individual experiences, ethical commitments, and challenges that arise, ranging from individual worker anxieties to allocation decision making regarding the distribution of limited resources to promote rehabilitation. The methodology will follow Yin’s [122] embedded single-case study methodology, wherein there is a single overarching case with 2 embedded units of analysis. The overarching case is the project, which includes the effort to generate and validate the SPARS technology, to co-design a program relying on that technology and to provide indications of acceptability and impact. The 2 embedded units of analysis will be (1) policy issues raised by SPARS technology and (2) the effort to co-design the rehabilitation program. The analysis will be open-ended, with no propositions guiding qualitative data collection, such that analysis can be as inductive as possible [122]. However, analytic insights will draw on the work of Hedgecoe [88,123] to gain insight into the appropriate balance between individual experiences and health system mandates, promoting a careful and critical analysis of the ethical issues outlined earlier.

#### Adherence-Driven Rehabilitation Program Design and Implementation

Adherence-driven rehabilitation and clinical management strategies will be codeveloped by our health sciences, bioengineering, and social sciences and humanities researchers and our principal Knowledge Translation User organization—leveraging input from the qualitative ethical and policy analysis—and our integrated knowledge translation activities. The design efforts will inform individual and program decision making on critical issues such as to whom and how the adherence analysis is presented and how adherence analysis should inform clinical decisions, physiotherapy treatment, resource allocation, and allied health provider engagement and support. Administration, clinician and patient user-interface, and workflow requirements will be confirmed through a user-centered design process detailed (subsequently) in our integrated knowledge translation strategy. The developed clinical process model and needs assessment will inform SPARS implementation from data collection to the presented analysis.

#### Pilot Test

The rehabilitation program will be pilot tested in 20 patients in the third year of research. The validated SPARS will provide the ability to monitor patient adherence to their prescribed home shoulder physiotherapy, allowing the treating physiotherapists and physicians to incorporate this information into their clinical decision making in accordance with the parameters of the program design. Baseline adherence predictors, inertial data, and clinical outcomes will be captured and stored as specified for the clinical validation study. Qualitative interviews will be conducted with patients, physicians, and stakeholders involved in the pilot program, for examination and analysis of postimplementation perspectives and challenges in accordance with Yin’s case study methodology [122]. Clinical end points and program cost-efficacy will be assessed in comparison with the matched historical controls involved in the validation study.

### Data Analysis

#### Sample Size

The proposed sample size of 120 for clinical validation yields a power of 80% to detect weak correlations (*R*>0.25) between physiotherapy adherence and clinical outcomes, accepting a 5% alpha error. Sample size feasibility was confirmed by the WCP, anticipating a recruitment rate of 70%. The pilot study sample size (20) was used to detect implementation challenges with an incidence rate greater than 15% at a 95% confidence level [124] (not statistically significant differences in clinical or economic outcomes).

#### Construct Validity

The construct validity of the SPARS adherence measures will be evaluated using cross-validated exercise type and technique classification performance metrics (accuracy, precision, recall, and f1-score) from the labeled inertial data. The cross-validated metrics will be assessed for 3 different fold splitting strategies: (1) by-subject; (2) by-session; and (3) within-session. Respectively, these estimate SPARS performance for (1) new subjects (with no training data); (2) new sessions for subjects with prior training data; and (3) new exercise repetitions within a session from which training data were acquired. The by-session cross-validation most closely approximates the use case in the proposed study and will benchmark SPARS performance against our stated measurable objectives.

#### Concurrent Validity

Concurrent validity of SPARS will be established with (1) patient home exercise adherence diaries; and (2) physiotherapist exercise logs recorded in the absence of the researchers. The number and type of exercises measured by SPARS will be evaluated against both the adherence diaries and physiotherapist logs. The evaluations of the SPARS technique will be compared with the physiotherapist logs. Systematic bias between measurement methods will be evaluated using a paired sample *t* test and Bland-Altman plots [125]. Reproducibility will be evaluated using an intraclass correlation with a 2-way random-effects model for absolute agreement interclass correlation coefficient (2,1) [126].

#### Criterion Validity

Changes in clinical outcomes will be evaluated as a function of SPARS adherence measures using univariate linear regression analysis, with management subgroup (conservative or operative) and population subgroup (worker or patient) as moderators. This analysis will examine both the concurrent and predictive effects of home physiotherapy adherence on clinical outcomes.

#### Inter-Rater Reliability

The inter-rater reliability of the exercise type and technique (human-assigned) labels will be evaluated using Fleiss kappa from the independent ratings of the treating physiotherapist recorded at the time of data collection and 2 research physiotherapists following a video review.

#### Univariable and Multivariable Analyses

The relationship between adherence (dependent variable) and individual baseline adherence predictor variables and environment (home or clinic) will be examined using parametric univariable statistical analyses, linear regression for continuous predictors, and 2-sample *t* tests and analysis of variance for binary and multiclass categorical predictors. A multivariable analysis will be conducted to model adherence from statistically significant predictors (maximum 12) and evaluate their relative importance.

#### Algorithmic Fairness

Equalized odds and equal opportunity fairness metrics [127] will be calculated for adherence classifiers on selected subgroups (sex and workers’ compensation status). Fairness-aware classifier training with prejudice regularization [128] will be considered if needed to produce fair results.

#### Economic End Point

The incremental cost-effectiveness ratio of the pilot program will be assessed by comparing patient health care resource utilization and return to work with matched historical controls [129].

## Results

### Approvals and Funding

This study was approved by the institutional research ethics board at the Sunnybrook Health Sciences Centre in Toronto, Ontario, Canada, on December 21, 2018. The project was successfully funded by the following 2 grant programs:

1. WSIB of Ontario Research Grants Program: January 1, 2019, to December 31, 2021.
2. Collaborative Health Research Project Special call: Artificial Intelligence, Health, and Society; Canadian Institutes of Health Research; Natural Sciences and Engineering Research Council; and Social Sciences and Humanities Research Council: April 1, 2019, to March 31, 2022.

### Timeline

**Figure 1:**
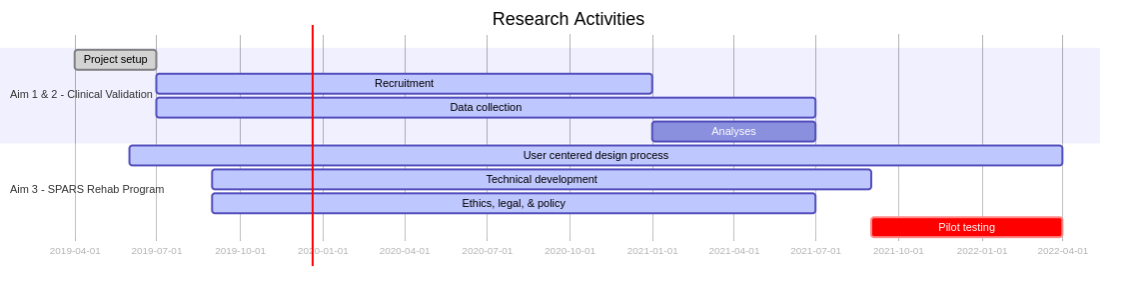
Study timeline. SPARS: Smart Physiotherapy Activity Recognition System.

The study timeline is shown in Figure 1. All activities are occurring on track as scheduled. Data collection will be completed by July 2021 and April 2022 for the clinical validation and implementation stages of the project, respectively.

### Recruitment

**Figure 2:**
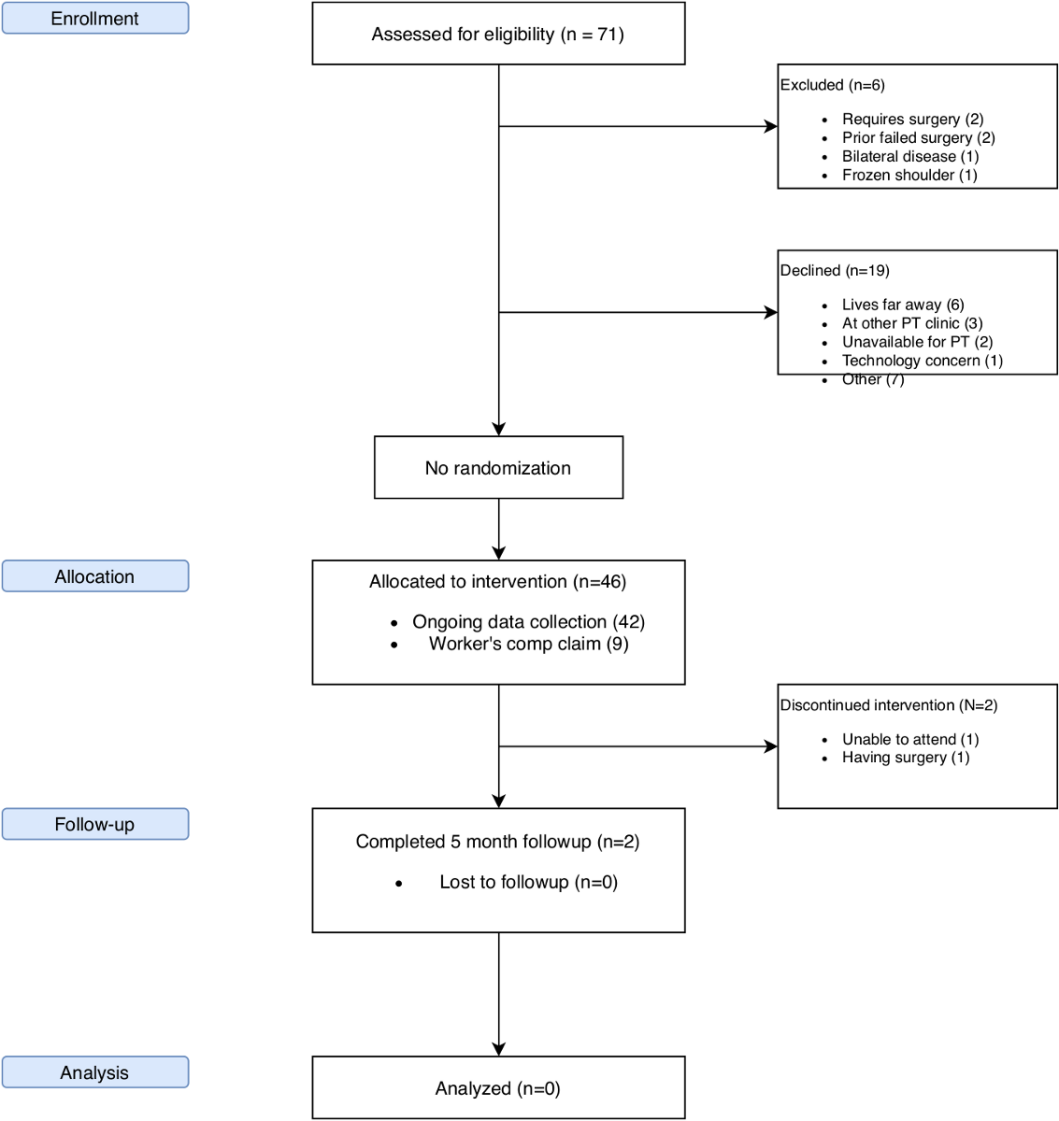
Recruitment and patient flow as of December 2019.

Patient recruitment began in July 2019. As of December 2019, 71 patients were screened for enrollment, of whom 65 patients met the inclusion and exclusion criteria. Of these, 46 patients consented and 19 declined to participate in the study. Of those recruited, 9 have worker’s compensation claims and 37 do not. Figure 2 shows the recruitment status and patient flow of the ongoing study.

### Data Collection App Details

Data collection is being performed using a custom Android Wear app and the Huawei Watch 2 smart watch. We are collecting 9-axis inertial sensor data and heart rate data during supervised and independent physiotherapy exercises. The sensor data are collected on the smart watch and uploaded to Google Cloud Storage servers. Labeling of the supervised activities is performed in a separate Android app running on an Android tablet (Samsung Tab A) and operated by our research team.

## Discussion

### Principal Findings

Exercise-based physiotherapy is considered essential in the management of rotator cuff pathology, both as a first-line treatment [4-6] and for postoperative rehabilitation [7,8]. The current paradigm for the delivery of physiotherapy services involves in-person patient assessments and training for prescribed exercise activities, but it requires the patients to perform majority of their exercises independently at home. There is a body of literature that indicates patients often struggle to fully engage in their prescribed activities [9,11], suggesting that many may not receive the full benefit of this important treatment.

A significant barrier to better understanding the impact of this problem on physiotherapy outcomes has been the challenge of accurately assessing adherence to physiotherapy participation in the home setting. This limitation precludes accurate and objective measurement of the administered physiotherapy dose for protocols that include an independent home component.

This manuscript describes a research protocol by which a smart watch and ML approach will be validated for tracking exercise-based shoulder physiotherapy in a cohort of patients with rotator cuff pathology. The validated instrument will also be used in a noninterventional fashion to assess the rate of adherence in the study population, the relationship between adherence and recovery, and patient factors related to physiotherapy adherence. As a final phase of this project, we will pilot an adherence-driven rehabilitation program where real-time measurement of physiotherapy adherence is utilized to promote better patient engagement in their home program.

A smart watch was selected as the platform for physiotherapy adherence tracking chiefly for patient comfort during exercise and ease of use. A significant proportion of the rotator cuff pathology demographic is elderly [16,17] and many have low technology literacy. The smart watch platform we have developed only requires that a patient put on their watch before physiotherapy exercise and that they ensure it is charged before doing so. This is in contrast to other systems that either require multiple sensors to be strapped to various points on the body [70] or a computer vision approach that requires some setup in the exercise environment to obtain and maintain an appropriate field of view [65]. In our setup, raw inertial data are processed using a modern deep learning approach, which we have shown previously to significantly outperform classical algorithms utilizing an engineered feature representation [15]. Although the deep learning approach requires significantly more computing resources for training, the inference time is comparable with classical models and feasible for evaluation either as a cloud service or directly on a patient’s mobile device [15].

There are a number of limitations to the proposed study. In the validation phase of this research, the act of measuring physiotherapy adherence may impact patients’ likelihood of adherence, even in a double-blinded noninterventional fashion, as proposed. Therefore, the assessed adherence rate in the study population may not be generalizable to populations that receive no tracking at all. A further limitation is that the ML algorithms validated in the clinic context will generalize well to the home setting. This risk will be mitigated by assessing algorithm generalization across multiple clinic sessions and to new patients (subject stratification). We assume that if that algorithm generalizes in both these cases, generalization to the home setting is also likely. As we also assess the criterion validity for adherence tracking, a positive relationship between adherence and recovery, if detected, it would serve as further evidence for the validity of algorithm construct.

Despite the efforts we have invested in developing a simple, accurate, and robust method for tracking shoulder physiotherapy, tracking errors will inevitably occur and result in imperfect adherence measurement. These errors could be secondary to software glitches, poor algorithm generalization, accidental or intentional misuse or disuse, hardware failure, and others. Therefore, the adherence estimates that we calculate for each patient will be subject to these errors, which may be random or biased to certain study subjects. To mitigate and assess these effects, we will collect survey data from each patient on their use of the smart watches and ask each patient to manually complete a 2-week adherence diary so that we can compare the measured adherence with patient self-report.

A limitation of the final interventional phase of this study is the pilot sample size (N=20) and the absence of a true control group. Although we can compare the interventional cohort with historical controls in the validation cohort, a better comparison would be with a control group with no tracking at all as per the current standard of care. Ultimately, a larger randomized controlled trial (RCT) would be required to adequately assess the effect of an adherence-driven rehabilitation program on recovery. The pilot study we conduct is primarily powered to detect implementation challenges, refine the system design, and estimate the effect size as required preliminaries to funding and carrying out a larger multicenter RCT.

### Conclusions

This protocol paper describes a peer-reviewed, grant-funded study to validate a smart watch and ML approach to assess patient adherence to exercise-based shoulder physiotherapy in a population with rotator cuff pathology. This study will provide new and important insights into shoulder physiotherapy adherence, the relationship between adherence and recovery, barriers to better adherence, and methods for addressing them. The ultimate goal of this work is to improve physiotherapy delivery and outcomes for patients with musculoskeletal injuries and disorders.

## Appendix

Multimedia Appendix 1 Peer review reports from Collaborative Health Research Projects - NSERC Partnered.

## Abbreviations

ANN: artificial neural networks
CRNN: convolutional recurrent neural network
DASH: Disabilities of the Arm, Shoulder, and Hand
IMU: inertial measurement unit
ML: machine learning
NPRS: Numeric Pain Rating Scale
RCT: randomized controlled trial
SPARS: Smart Physiotherapy Activity Recognition System
WCP: Working Conditions Program
WSIB: Workplace Safety and Insurance Board
