# Supplementary material for: Adherence Tracking With Smart Watches for Shoulder Physiotherapy in Rotator Cuff Pathology: Protocol for a Longitudinal Cohort Study
Source: JMIR Res Protoc. 2020 Jul 5;9(7):e17841. doi: 10.2196/17841 (PMC7381014; doi:10.2196/17841)
Supplement: Multimedia Appendix 1 [file resprot_v9i7e17841_app1.pdf]

April/Avril 01, 2019

DC0190GP

Canadian Institutes of Health Research / Instituts de recherche en santé du Canada

Notice of Decision / Avis de décision

Application Number/Numéro de la demande: 414807

Committee Code/Code du comité: DAN

Applicants/Candidats: Dr. Cari Marisa Whyne   Prof. Robin R Richards   Dr. Helen Razmjou

With/Avec: Dr. D. Burns   Dr. J. Shaw

Institution paid/ Établissement payé: University of Toronto

Title/Titre: Shifting the Paradigm in Home Physiotherapy: Implementation and Implications of Adherence Monitoring with Artificial Intelligence

Primary Inst./ Inst. principal: Musculoskeletal Health and Arthritis / Appareil locomoteur et arthrite

Other Related Inst./ Autres inst. connexes: Health Services and Policy Research / Services et politiques de la santé; Aging / Vieillissement

Competition Outcome/Résultats du concours: Collaborative Health Research Projects (NSERC partnered) / Prog de projets rech concertée sur la santé (en partenariat avec le CRSNG)

October/Octobre 30, 2018

Number in competition/Nbre de demandes dans le concours: 98

Number approved/Nbre de demandes approuvées: 29

Decision on your application/ Décision sur votre demande: Approved / Approuvée

Average annual amount/ Montant annuel moyen: $107,441

Equipment amount/ Montant pour les appareils: $25,000

Term/Durée: 3 **yrs/ans** 0 **months/mois**

Peer Review Committee Recommendation, for your information and use/
Recommandation du comité d'examen par les pairs, pour fins d'information et d'utilisation:

Committee/Comité: Collaborative Health Research Projects - NSERC Partnered / Projets de recherche concertée sur la santé - en partenariat avec le CRSNG

Application rank within the competition/ Rang de la demande dans ce concours: 24

Percent Rank Within the Competition/ Rang en pourcentage au sein du concours: 24.49%

Rating/ Cote:

Scientific Merit	4.07
Potential Impact	4.08

Recommended average annual amount/ Montant annuel moyen recommandé: $223,215

Recommended equipment amount/ Montant recommandé pour les appareils: $25,000

*** Applications receiving a score of less than 3.5 on any evaluation criteria will not be considered for Funding. / Les demandes qui ont reçu une note inférieure à 3.5 pour n'importe quel des critères d'évaluation ne sont pas admissibles.

This document is for information only. Official payment is stated on the CIHR Authorization for Funding.
Document à titre d'information seulement. Le paiement officiel est indiqué sur l'autorisation de financement des IRSC.

Canadian Institutes of Health Research
Instituts de recherche en santé du Canada

160 Elgin Street, 9th Floor
Address Locator 4809A
Ottawa, Ontario K1A 0W9

160, rue Elgin, 9e étage
Indice de l'adresse 4809A
Ottawa (Ontario) K1A 0W9

Institute of Aging
Institute of Cancer Research
Institute of Circulatory and Respiratory Health
Institute of Gender and Health
Institute of Genetics
Institute of Health Services and Policy Research
Institute of Human Development, Child and Youth Health
Institute of Indigenous Peoples' Health
Institute of Infection and Immunity
Institute of Musculoskeletal Health and Arthritis
Institute of Neurosciences, Mental Health and Addiction
Institute of Nutrition, Metabolism and Diabetes
Institute of Population and Public Health

Institut du vieillissement
Institut du cancer
Institut de la santé circulatoire et respiratoire
Institut de la santé des femmes et des hommes
Institut de génétique
Institut des services et des politiques de la santé
Institut du développement et de la santé des enfants et des adolescents
Institut de la santé des Autochtones
Institut des maladies infectieuses et immunitaires
Institut de l'appareil locomoteur et de l'arthrite
Institut des neurosciences, de la santé mentale et des toxicomanies
Institut de la nutrition, du métabolisme et du diabète
Institut de la santé publique et des populations

April 1, 2019

Cari Whyne
Sunnybrook Research Institute
2075 Bayview Avenue, S-620
Toronto, ON M4N 3M5

Dr. Whyne:

On behalf of the Canadian Institutes of Health Research (CIHR) in partnership with the Natural Sciences and Engineering Research Council of Canada (NSERC) and in collaboration with the Social Sciences and Humanities Research Council (SSHRC), we are pleased to inform you that your recent application entitled "*Shifting the Paradigm in Home Physiotherapy: Implementation and Implications of Adherence Monitoring with Artificial Intelligence*", submitted to the Collaborative Health Research Projects funding opportunity, has been approved.

Since you are receiving this letter through ResearchNet, you will also find posted the competition results and your application reviews. As CIHR does not notify other principal applicants or co-applicants of the decision taken, we ask that you please inform those individuals involved, along with their research institutions (if different from your own), of the outcome of this application. Additional supporting documents from both CIHR and NSERC will follow in the mail shortly.

Should you have any questions about the review process, please address them directly with CIHR staff. Do not contact the officers or members of the peer review committee.

Congratulations on your success in this competition. Should you have any questions or concerns, do not hesitate to contact CIHR's Contact Centre (telephone: 613-954-1968; email: support@cihr-irsc.gc.ca).

Sincerely,

Nathalie Gendron Ph.D.
Manager, Competition Delivery
Priority Driven Research
CIHR

Jennifer Mills
Manager,
Research Partnerships Programs
NSERC

Wafa Bitar
Manager
Research Grants and Partnerships Division
SSHRC

cc: NSERC, SSHRC

**Application Number / Numéro de demande:**	414807
**Name of Applicant / Nom du chercheur:**	Whyne, Cari Marisa
**Review Type / Type d'évaluation:**	Committee Member 1/Membre de comité 1
**Competition:**	2018-10-30 Collaborative Health Research Projects (NSERC partnered)
**Concours:**	2018-10-30 Programme de projets de recherche concertée sur la santé (en partenariat avec le CRSNG)
**Committee:**	Collaborative Health Research Projects - NSERC Partnered
**Comité:**	Projets de recherche concertée sur la santé - en partenariat avec le CRSNG

Potential Impact

Comments:

Shoulder problems are a common issue and there is little evidence that surgical treatment is any better than rehabilitation. Monitored home treatment programs are a promising option for the treatment of shoulder pathology but measuring and then addressing adherence to the program remains a challenge. Measurement of adherence will enable a focussed approach for evaluating home programs as higher adherence to an effective program will improve outcomes.

Dr. Richards of Sunnybrook Working Conidition Program (WCP) is the primary knowledge user. The WCP treats thousands of patients and injured workers every year.

Trainees working on this project will be immersed in a highly integrated NSE and Health Sciences environment. One PhD, one postdoc, and two co-op students will be trained. The PhD student will focus on developing SPARS, the health science postdoc and MSc student will work on qualitative clinical assessments and ethics. A surgeon-scientist will also be trained. The lab environment emphasizes bench to bedside research and has a strong track record training students in this area.

The KT plan starts at the T2, translation to patients phase. The Working Condition Program (WCP) will provide access to patients and injured workers. Once a clinical solution is developed the SPARS system will be commercialized using the established pipelines at Sunnybrook. A provisional patent is already being filed for the preliminary work. The KT will provide $10K in cash as well as $90K of In-Kind from their Physios which indicates a very strong commitment to the project’s success. They plan to use the outputs of this research to support their application to register with Health Canada and FDA as a Class I Medical Device.

**Application Number / Numéro de demande:**	414807
**Name of Applicant / Nom du chercheur:**	Whyne, Cari Marisa
**Review Type / Type d'évaluation:**	Committee Member 1/Membre de comité 1
**Competition:**	2018-10-30 Collaborative Health Research Projects (NSERC partnered)
**Concours:**	2018-10-30 Programme de projets de recherche concertée sur la santé (en partenariat avec le CRSNG)
**Committee:**	Collaborative Health Research Projects - NSERC Partnered
**Comité:**	Projets de recherche concertée sur la santé - en partenariat avec le CRSNG

Scientific Merit

Comments:

This proposal aims to develop a smart physiotherapy activity recognition system (SPARS) for tracking home physiotherapy exercises using sensors embedded in a watch. The system will use AI algorithms that were demonstrated to be effective during a pilot test on healthy individuals. The first target of the system is to measure adherence to home therapy programs and the second goal is to assess the quality of movement. The investigators also plan to develop an ‘ethically conscious’ rehab program based on their technology.

This is a very well written and organized proposal – broken into three aims. In Aim 1 the investigators will develop and validate the SPARS technology for home use. Data will be captured on 60 injured workers and 60 patients. This data will be used to train the AI with targets of 90% for classifying (differentiating) different activities and 80% for assessing technique or quality of movement.

In Aim 2 the investigators will measure the rate of adherence and examine the relationship between adherence and recovery. The investigators test three hypotheses here (technique and adherence is better for supervised sessions, participation and outcomes will be correlated, and these factors will be related to workers comp. Aim 3 will develop the shoulder rehab program. The approach will begin with interviews with stakeholders, develop rehab strategies that integrate with the SPARS system and will pilot the work on 10 patients and 10 injured workers.

Strengths of this proposal include the substantial pilot work demonstrating proof of concept, the amount of detail and demonstrated use of the machine learning and AI methodology, the systematic approach to develop and implement rehabilitation protocols that can be monitored with the SPARS system, and the ethical considerations.

The team is very strong. Dr. Whyne is a bioengineer with experience conducting interdisciplinary work. Dr. Burns and Fetaya bring machine learning, while Dr. McLachlin and Hardisty bring expertise in wearable technology and software / data management respectively. Dr. Razmjou is a physiotherapist, clinician-researcher and Dr. Henry is in orthopaedic surgery. Dr. Shaw is a social sciences researcher with expertise on qualitative methods around health care delivery.

**Application Number / Numéro de demande:**	414807
**Name of Applicant / Nom du chercheur:**	Whyne, Cari Marisa
**Review Type / Type d'évaluation:**	Committee Member 2/Membre de comité 2
**Competition:**	2018-10-30 Collaborative Health Research Projects (NSERC partnered)
**Concours:**	2018-10-30 Programme de projets de recherche concertée sur la santé (en partenariat avec le CRSNG)
**Committee:**	Collaborative Health Research Projects - NSERC Partnered
**Comité:**	Projets de recherche concertée sur la santé - en partenariat avec le CRSNG

Potential Impact

Comments:

Project is to implement automated measures of shoulder exercises to improve adherence.
Impact is to improve home physio by doing exercises correctly.
First aim is to develop ML algorithms to detect what exercise the user is doing, based on data from the sensors in an iwatch.
But it isn't known if adhering to the exercises will actually improve outcome, so the project has a big risk.

KTranslation will be through Sunnybrook working condition program.
Clinical outcomes: return to work status, shoulder range of motion, rotator cuff strength, and patient reported outcomes of pain and disability are collected pre-treatment, monthly through treatment (up to 5 months), and at 12 months final follow-up. Final follow-up exceeds 85%.

Ultimate goal: "The researchers will leverage the well-established pipeline for clinical translation of new technology at SRI with expected commercialization through a commercial partnership, software licensing agreement, or start-up company."
This is very vague--the KT transfer is not as obvious as for other projects.

Furthermore, there is training for only 1 PDF, 1PhD and 1MSc for a large budget--much of the money will go towards research coordinators, a clinical RA full time, and physiotherapists.

**Application Number / Numéro de demande:**	414807
**Name of Applicant / Nom du chercheur:**	Whyne, Cari Marisa
**Review Type / Type d'évaluation:**	Committee Member 2/Membre de comité 2
**Competition:**	2018-10-30 Collaborative Health Research Projects (NSERC partnered)
**Concours:**	2018-10-30 Programme de projets de recherche concertée sur la santé (en partenariat avec le CRSNG)
**Committee:**	Collaborative Health Research Projects - NSERC Partnered
**Comité:**	Projets de recherche concertée sur la santé - en partenariat avec le CRSNG

Scientific Merit

Comments:

Project is to determine home physiotherapy adherence monitoring after shoulder injury/surgery using inertial sensors in a smart watch. Compare supervised vs unsupervised excercises.

Physical therapy is essential for the successful rehabilitation of common shoulder injuries and following shoulder surgery. Patients may receive some training and supervision for shoulder physiotherapy through private pay or private insurance, but they are typically responsible for performing most of their physiotherapy independently at home. It is unknown how often patients perform their home exercises and if these exercises are done correctly without supervision. There are no established tools for measuring this. It is therefore unclear if the full benefit of shoulder physiotherapy treatments are being realized.
The proposed study seeks to establish if there are kinematic differences between supervised and home rotator cuff rehabilitation exercise, and determine if there is a relationship between exercise technique and shoulder recovery.
Existing Innovative Smart Physiotherapy Activity Recognition System SPARS technology, identified shoulder excercises in 20 healthy adults--will be further validated on 120 patients (60 with WCB claim, and 60 without) and tested in this project.
The research is to classify physiotherapy exercise type and technique (from the i-watch sensor data), and then also determine if adhering to the supervised exercises actually improves the outcome.

The ML/AI in the proposal is to use the sensor data to determine the exercises.
They already have made pilot measurements on 20 healthy volunteers, and wish to extend to 120 patients.
This is a very large budget to extend the trained neural net to sufficient accuracy on patients (milestone 5) and this shouldn't take 2 years.

Final year, 10 patients and 10 injured workers will test the system.
Good power statistics for how many subjects needed.

**Application Number / Numéro de demande:**	414807
**Name of Applicant / Nom du chercheur:**	Whyne, Cari Marisa
**Review Type / Type d'évaluation:**	Committee Member 3/Membre de comité 3
**Competition:**	2018-10-30 Collaborative Health Research Projects (NSERC partnered)
**Concours:**	2018-10-30 Programme de projets de recherche concertée sur la santé (en partenariat avec le CRSNG)
**Committee:**	Collaborative Health Research Projects - NSERC Partnered
**Comité:**	Projets de recherche concertée sur la santé - en partenariat avec le CRSNG

Potential Impact

Comments:

This project aims to develop a Smart Physiotherapy Activity Recognition System (SPARS) to monitor patient’s adherence to their physiotherapy treatment at home, following rotator cuff pathology. The applicants intend to develop this system using new wearable technologies and machine learning (ML) techniques to process the data collected with the wearable devises. Once operational, the system should help establish if patients are performing their physiotherapy exercises correctly. The project is structure around three aims: Aim (1) is to develop and validate SPARS for evaluating home shoulder physiotherapy adherence. Aim (2) is to measure the rate of adherence to home shoulder physiotherapy, the relationship between adherence and recovery, and identify barriers to home physiotherapy adherence. Aim (3) is to develop and pilot test a conscientious SPARS-powered shoulder rehabilitation program that provides individualized adherence-driven patient care in accordance with ethical innovation and user-centered design practices.

The project may have an important impact from a clinical perspective in allowing attending physicians to establish if patients are conforming with physiotherapy treatments. Furthermore, this may help to clarify the relationship between recovery from shoulder injury with the rate and quality of participation with home shoulder therapy. According to the applicants, there is very few reliable data on patient conformity with physiotherapy treatments at this point.

However, the applicants could provide more explanations on what they intend to do within this project from an ethical or policy perspective. They explain that the proposed project will raise a number of ethical issues: first, patient surveillance may influence the behavior of participants (an issue also known as the Hawthorne effect). Second, there will be questions of just resource-allocation if more resources are allocated to patient that are not able to conform with physiotherapy treatment at home. Third, biases could be built in the algorithm itself. Then, they claim that these points will be addressed in the project through an ethical or policy analysis, but they do not provide clear explanations on what this analysis will entail.

The method section in the research proposal also explains that the applicants will conduct complete qualitative interviews with patients, health care providers and policy-level stakeholders “to examine different perspectives on individual experiences, ethical commitments and challenges that arise ranging from individual worker anxieties to allocation decision-making regarding the distribution of limited resources to promote rehabilitation.” This is useful information, but the objectives of this initiative, its underlying hypothesis or its main research question are still not clear. Therefore, it is difficult to provide a reliable assessment of the potential impact of the project as well as training opportunities or KT opportunities.

**Application Number / Numéro de demande:**	414807
**Name of Applicant / Nom du chercheur:**	Whyne, Cari Marisa
**Review Type / Type d'évaluation:**	Committee Member 3/Membre de comité 3
**Competition:**	2018-10-30 Collaborative Health Research Projects (NSERC partnered)
**Concours:**	2018-10-30 Programme de projets de recherche concertée sur la santé (en partenariat avec le CRSNG)
**Committee:**	Collaborative Health Research Projects - NSERC Partnered
**Comité:**	Projets de recherche concertée sur la santé - en partenariat avec le CRSNG

Scientific Merit

Comments:

The project raises interesting ethical and policy questions, but it is not clear how these questions will be addressed, and what are the precise objectives, hypothesis or research questions under aim (3) of the project (see my comments in the previous section). Therefore, I would need to have more explanations before I can conclude that the project has sufficient scientific merit regarding its ethical and public policy dimension.

Review Type/Type d'évaluation: SO Notes /Notes de l'agent scientifique
Name of Applicant/Nom du chercheur: Whyne, Cari Marisa
Application No./Numéro de demande: 414807
Agency/Agence: CIHR/IRSC
Competition/Concours: 2018-10-30 Collaborative Health Research Projects (NSERC partnered)/Programme de projets de recherche concertée sur la santé (en partenariat avec le CRSNG)
Committee/Comité: Collaborative Health Research Projects - NSERC Partnered/Projets de recherche concertée sur la santé - en partenariat avec le CRSNG
Title/Titre: Shifting the Paradigm in Home Physiotherapy: Implementation and Implications of Adherence Monitoring with Artificial Intelligence

---

Assessment/Évaluation:

Shifting the paradigm in home physiotherapy: Implementation and implications of adherence monitoring with artificial intelligence

- Points raised

• Strong group of researchers and an appropriate knowledge user, with the KU making strong contributions and having a clear goal for translation. The project brings together a number of established researchers and approaches from different disciplines.

• Approach is clear and has strong potential for impact; however, there was not enough information provided regarding the ethical and policy concerns related to the project. For example, biases can be built into algorithms, and patients' behaviour can change when they are being monitored. In general, the ethical and policy issues were under-developed.

• It also is not known if individuals who adhere to exercise will have improved outcomes, which is a risk to the project.

• Training plan is strong. The applicants have a strong record of training, and there will be good interdisciplinary interactions among trainees.

• SGBA was addressed well.

- Budget - ok
- Term - ok
